# Alveolar soft-part sarcoma with lymph node metastasis in the rectum

**DOI:** 10.1055/a-2503-6056

**Published:** 2025-01-14

**Authors:** Youming Xu, Huihua He, Qiuyun Yang, Wei Tan

**Affiliations:** 1 117921 Department of Gastroenterology, Renmin Hospital of Wuhan University, Wuhan, China; 2 117921 Department of Pathology, Renmin Hospital of Wuhan University, Wuhan, China; 3 Department of Gastroenterology, Xianfeng County Traditional Chinese Medicine Hospital, Wuhan, China

A 37-year-old woman was referred to our department because of prolonged abdominal pain and anemia. She underwent a colonoscopy, which revealed a 12-mm protuberance in the rectum ( Fig. 1 **a** ). In addition, an MRI scan showed a 37-mm mass within the mesorectal envelope ( Fig. 1 **b** ). Based on these clinical and imaging features, a rectal tumor and enlarged extramural lymph node were suspected. Subsequently, endoscopic ultrasonography (EUS) was performed and revealed two contrasting hypoechoic lesions ( Video 1 ). The smaller, intraluminal lesion involved the 1st to 3rd layers of the rectum and had partially indistinct boundaries with the muscularis propria, which appeared slightly thickened ( Fig. 2 ). The larger lesion showed minimal blood flow and was considered to be an enlarged lymph node. An EUS-guided biopsy was then conducted for evaluation. No biopsy was performed on the intrarectal lesion due to its small size. Microscopic examination indicated focal myofibroblast and small-vessel proliferation, consistent with a benign mesenchymal tumor. However, immunohistochemistry ruled out gastrointestinal stromal tumor, schwannoma, and leiomyoma. Unfortunately, cytology and liquid-based cytology tests detected malignant adenocarcinoma cells. Ultimately, the patient underwent Dixon surgery and lymphadenectomy. Metastases were observed in 3 of the 23 mesenteric lymph nodes, with the largest lesion measuring 3.5 cm in diameter. Radiotherapy was performed 1 week after surgery. Postoperative specimens gave positive results on ASPSCR1/TFE3 gene testing, with fusion of the ASPSCR1 and TFE3 genes, and on TFE3 (Xp11.2) gene testing, with evidence of TFE3 gene fragmentation ( Fig. 3 ).

**Fig. 1:**
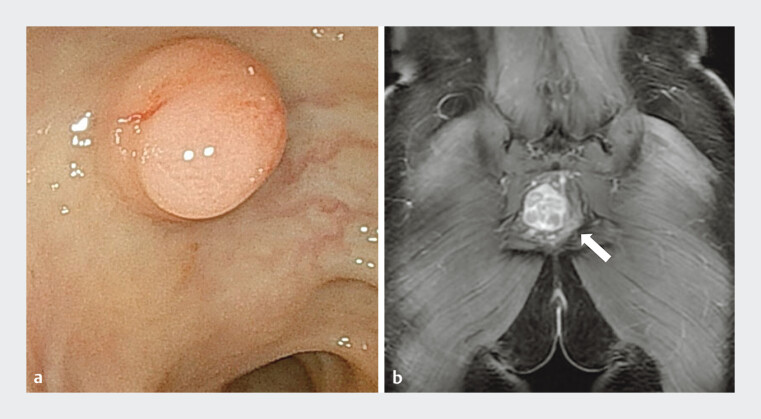
Rectal wall with two contrasting lesions: one internal, one external. **a** The primary lesion (approximately 10 mm) in the rectal lumen. **b** A 37-mm mass within the mesorectal envelope.

**Fig. 2:**
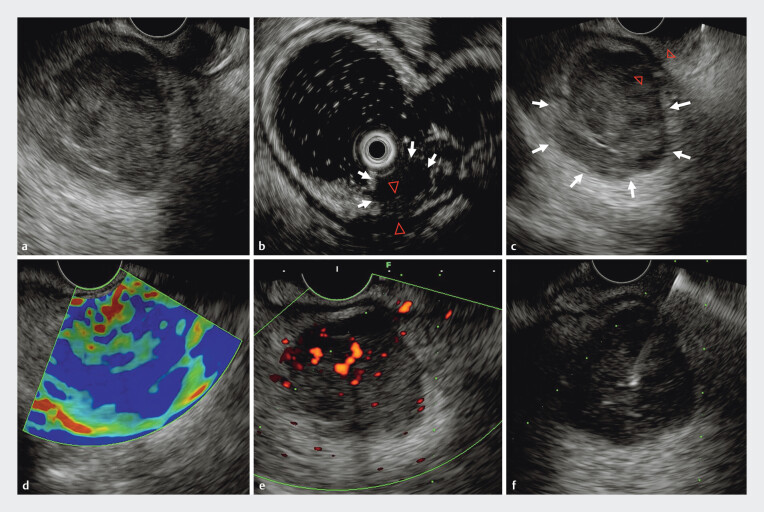
Endoscopic ultrasound (EUS) images. **a** Two hypoechoic contrasting lesions detected by EUS. **b** The primary hypoechoic lesion measured 9.49×7.75 mm on EUS and involved the 1st to 3rd layers of the rectum; its boundaries with the muscularis propria were partially indistinct. **c** The metastatic hypoechoic lesion measured 31.7×28.3 mm on EUS; it was observed outside the rectal wall and had partially indistinct boundaries with the muscularis propria. **d** EUS elastography showed the metastatic lesion with a blue-green pattern, predominantly blue. **e** The metastatic lesion exhibited minimal blood flow signals. **f** Endoscopic ultrasound-guided fine-needle aspiration was performed.

**Fig. 3:**
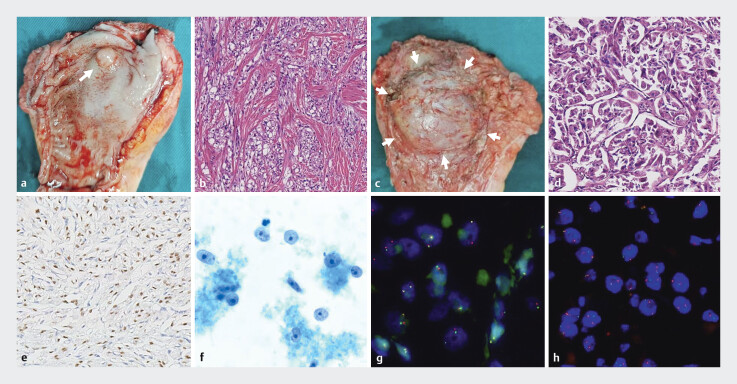
Pathological findings. **a** The primary lesion in the rectal lumen. **b** The hemangiopericytic capsule surrounding the tumor cells in the blood vessels. **c** The huge mass within the mesorectal envelope. **d** Alveolar-like tissue structure. **e** TFE3 immunostaining (diluted at 1:1; Cell Marque, Rocklin, California, USA). **f** Liquid-based cytology tests detected malignant adenocarcinoma cells. **g** The TFE3 (Xp11.2) gene test result is positive, indicating a break in the TFE3 gene. **h** The ASPSCR1/TFE3 gene test result is positive, indicating fusion of the ASPSCR1 and TFE3 genes.

Alveolar soft-part sarcoma (ASPS), a rare young adult sarcoma, often metastasizes early. This unique case of rectal ASPS with lymph node metastasis was differentiated from gastrointestinal submucosal tumors and diagnosed via endoscopic ultrasonography.Video 1

To the best of our knowledge, this is the first report of primary alveolar soft-part sarcoma (ASPS) in the rectum with lymph node metastases. Diagnosing ASPS in the gastrointestinal (GI) tract is challenging because of the variety of symptoms, limited availability of specimens, and similarities with poorly differentiated carcinoma 1 . While endoscopic mucosal biopsy is used for mucosal GI ASPS, submucosal lesions require EUS for tissue acquisition 2 . GI ASPS may present as raised adenoma-like lesions or submucosal masses, and differential diagnosis versus other GI tumors is necessary 3 . In our patient, EUS-guided fine-needle aspiration confirmed that the lesions in the rectal lumen and the mesorectal lymph node originated from the same site, identified as metastatic. TNM staging was definitively determined as T1N1M0, with endoscopic ultrasonography and fine-needle aspiration playing a pivotal role in the assessment.

Endoscopy_UCTN_Code_CCL_1AD_2AC

## Notes

Endoscopy E-Videos https://eref.thieme.de/e-videos
*E-Videos* is an open access online section of the journal *Endoscopy* , reporting on interesting cases and new techniques in gastroenterological endoscopy. All papers include a high-quality video and are published with a Creative Commons CC-BY license. Endoscopy E-Videos qualify for HINARI discounts and waivers and eligibility is automatically checked during the submission process. We grant 100% waivers to articles whose corresponding authors are based in Group A countries and 50% waivers to those who are based in Group B countries as classified by Research4Life (see: https://www.research4life.org/access/eligibility/ ). This section has its own submission website at https://mc.manuscriptcentral.com/e-videos .
